# 3D Printing of Solution‐Processable 2D Nanoplates and 1D Nanorods for Flexible Thermoelectrics with Ultrahigh Power Factor at Low‐Medium Temperatures

**DOI:** 10.1002/advs.201901788

**Published:** 2019-10-14

**Authors:** Chaochao Dun, Wenzheng Kuang, Nicholas Kempf, Mortaza Saeidi‐Javash, David J. Singh, Yanliang Zhang

**Affiliations:** ^1^ Department of Aerospace and Mechanical Engineering University of Notre Dame Notre Dame IN 46556 USA; ^2^ Department of Physics and Astronomy University of Missouri Columbia MO 65211 USA

**Keywords:** 1D/2D nanocrystals, 3D aerosol jet printing, flexible thermoelectrics

## Abstract

Solution‐processable semiconducting 2D nanoplates and 1D nanorods are attractive building blocks for diverse technologies, including thermoelectrics, optoelectronics, and electronics. However, transforming colloidal nanoparticles into high‐performance and flexible devices remains a challenge. For example, flexible films prepared by solution‐processed semiconducting nanocrystals are typically plagued by poor thermoelectric and electrical transport properties. Here, a highly scalable 3D conformal additive printing approach to directly convert solution‐processed 2D nanoplates and 1D nanorods into high‐performing flexible devices is reported. The flexible films printed using Sb_2_Te_3_ nanoplates and subsequently sintered at 400 °C demonstrate exceptional thermoelectric power factor of 1.5 mW m^−1^ K^−2^ over a wide temperature range (350–550 K). By synergistically combining Sb_2_Te_3_ 2D nanoplates with Te 1D nanorods, the power factor of the flexible film reaches an unprecedented maximum value of 2.2 mW m^−1^ K^−2^ at 500 K, which is significantly higher than the best reported values for p‐type flexible thermoelectric films. A fully printed flexible generator device exhibits a competitive electrical power density of 7.65 mW cm^−2^ with a reasonably small temperature difference of 60 K. The versatile printing method for directly transforming nanoscale building blocks into functional devices paves the way for developing not only flexible energy harvesters but also a broad range of flexible/wearable electronics and sensors.

## Graphical abstract

Solution‐processed 2D and 1D nanostructures are directly transformed into flexible thermoelectric devices using a scalable 3D conformal printing approach. The power factor of the nanocomposite film consisting of Sb_2_Te_3_ nanoplates and Te nanorods reaches an ultrahigh value of 2.2 mW m^−1^ K^−2^ at 500 K. A printed device produces a competitive power density of 7.65 mW cm^−2^ with a 60 K temperature difference.

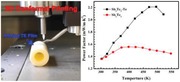

## 1 Introduction

Flexible thermoelectric generators (*f*‐TEGs) based on materials,^1^, ^2^, ^3^, ^4^, ^5^, ^6^ such as Sb_2_Te_3_ and Bi_2_Te_3_,^7^, ^8^ carbon nanotube,^9^ graphene,^10^ conductive polymer, and hybrids,^11^ have the ability to interconvert thermal to electrical energy without moving parts. These *f*‐TEGs can be integrated with portable/wearable electronics and sensors, and enable self‐powered devices. In this context, V_2_–VI_3_ metal chalcogenides (Bi_2_Te_3_, Sb_2_Te_3_, and related alloys and compounds)^1^, ^12^, ^13^, ^14^, ^15^, ^16^, ^17^ have attracted particular attention because of their high figure of merit (*ZT*) near room temperature.^18^ For example, p‐type Bi_2_Te_3_‐Sb_2_Te_3_ alloys show high performance near room temperature and benefit considerably from nanostructuring.^19^

Similar to Bi_2_Te_3_, Sb_2_Te_3_ is also a topological insulator,^20^ which leads to a complex, nonparabolic band structure, often highly favorable for thermoelectric (TE) performance.^21^ It has an extremely high dielectric constant of ε_0_ ≈ 100, favorable for high mobility even with large concentration of defects.^22^, ^23^, ^24^ Thus Sb_2_Te_3_ is potentially an important TE material, the key challenge being to find methods to control its carrier concentration and to effectively nanostructure the material while maintaining this control. So far, most of the reported Sb_2_Te_3_ related materials are p‐type semiconductors. This is caused by intrinsic defects including Sb vacancies and antisite defects of Sb atoms on the Te sites (Sb_Te_)^25^ that occur during normal synthesis procedures. Typically, Sb_2_Te_3_ bulk single crystals stand out for their unique advantages including a high electrical conductivity (σ) around 3–5 × 10^5^ S m^−1^, and a reasonable thermal conductivity (κ) around 1–6 W m^−1^ K^−1^. However, Sb_2_Te_3_ also has a less competitive Seebeck coefficient (*S*) around 83–105 µV K^−1^. This is due to its high degenerate hole concentration (*n* > 10^20^ cm^−3^) created by the acceptor states mentioned above,^26^ especially Sb_Te_. Thus key problem is to find ways to control the doping level and thereby reduce the hole concentration and to determine the extent to which this can lead to enhanced *S* and TE performance. Nanostructuring has been employed to enhance *S*, and to reduce κ as a result of the increased phonon scattering effect.^19^, ^27^, ^28^, ^29^, ^30^ For example, Sb_2_Te_3_ with 2D nanoplates morphology presents a 30% increase in *S* (*S* = 125 µV K^−1^) near room temperature.^31^ *S* and *ZT* enhancement in nanostructured Sb_2_Te_3_ by antisite defect suppression through sulfur doping was also achieved in nanobulk thermoelectrics.^32^ Bi‐Te and Sb‐Te solid solutions (e.g., Bi_0.5_Sb_1.5_Te_3_) also increase *S* (*S* > 170 µV K^−1^ at 450 K) and suppress κ. Unfortunately, the reduced bandgap limits the ability of the Bi‐Sb‐Te system to retain high *ZT* above 450 K.^33^, ^34^ Despite the high *ZT* (>1) observed in the Bi‐Sb‐Te system, the *ZT* normally peaks at narrow temperature range near or below 100 °C.^19^, ^35^, ^36^, ^37^, ^38^ It has been an exacting challenge to develop materials with consistently high *ZT* with broad temperature plateau.^39^, ^40^ Meanwhile, there is also an absence of high *ZT* materials in the middle temperature range (200–300 °C) where the majority of the waste heat resides.^27^

Although TE nanostructures with enhanced *ZT* have been extensively studied, a big gap exists in translating these nanostructures into high‐performance and flexible TE devices. The majority of reported TE devices were fabricated by exploiting the above‐mentioned inorganic nanostructures into bulk configurations.^41^, ^42^ However, rigid bulk devices not only consume large amount of TE materials but also present challenges in applications on curved surfaces such as the human body or even exhaust pipes.^15^, ^43^ We previously demonstrated an ultrafast photonic sintering method to process aerosol jet printed flexible Bi_2_Te_2.7_Se_0.3_ n‐type films. This led to a good room‐temperature power factor (PF) of 0.73 mW m^−1^ K^−2^.^17^ While there have been recent advancements in flexible n‐type films with competitive PF,^1^, ^6^ thermoelectrics need both p‐type and n‐type materials. The corresponding PF values for the flexible p‐type films remain below or around 1 mW m^−1^ K^−2^ (Table S4, Supporting Information). In this work, a flexible p‐type film with ultrahigh PF was fabricated based on a novel hybrid ink formulation consisting of 1D/2D nanostructures using a highly scalable 3D conformal aerosol jet printing method. The nanocomposite film printed using 2D Sb_2_Te_3_ nanoplates and 1D Te nanorods demonstrated a PF of 1.36 mW m^−1^ K^−2^ at room‐temperature and a peak PF of 2.2 mW m^−1^ K^−2^ at 500 K, which fills in a gap of the absence of high‐performance and cost effective TE materials in the low‐medium temperature range around 400–500 K.^44^ In addition, we report theoretical work that elucidates the fundamental mechanisms leading to the enhanced power factor in this hybrid 2D/1D material. A printed flexible TE generator exhibits a high power density of 7.65 mW cm^−2^ under a small temperature gradient of 60 K, demonstrating great potential to power wearable electronics and sensors.

## 2 Results and Discussion

**Figure 1:**
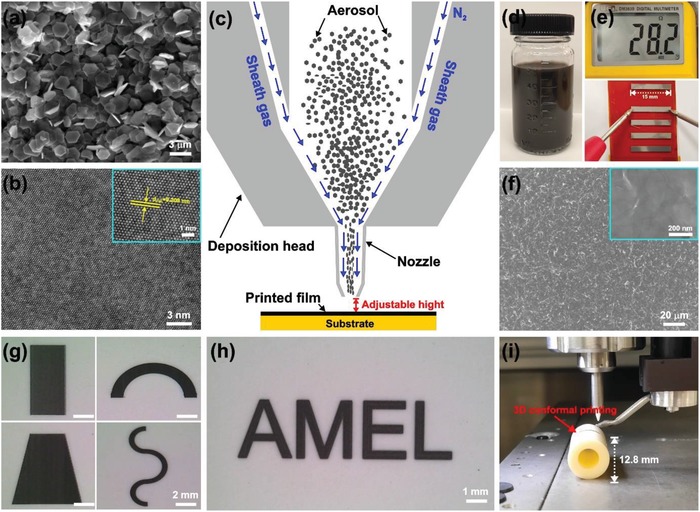
a) SEM and b) HRTEM images of Sb_2_Te_3_ nanoplates. c) Schematic image of 3D conformal aerosol jet printing process using solution‐processed nanostructures as building blocks. d) Synthesized Sb_2_Te_3_ ink with high stability that is printable for at least 3 months. e) Typical printed thin films with internal resistance around 30 Ω. f) SEM image of Sb_2_Te_3_ film on polyimide after sintering at 400 °C for an hour. Films with designed patterns printed on various substrates: g) printed TE legs with different shapes, h) pattern with “AMEL” design, and i) 3D conformal printed film on a curved surface.

Sb_2_Te_3_ nanoplates were fabricated using a facile energy‐saving hydrothermal method (see details in the Experimental Section). **Figure** **1**a,b shows the scanning electron microscopy (SEM) and high‐resolution transmission electron microscopy (HRTEM) images of the 2D Sb_2_Te_3_ nanoplates with high crystallization. The lattice spacing of 0.209 nm corresponds to the lattice planes of (110) in Sb_2_Te_3_. Figure S1 (Supporting Information) presents the powder X‐ray diffraction (XRD) pattern of the as‐fabricated Sb_2_Te_3_ plates, together with the major diffraction peaks which correspond to rhombohedral Sb_2_Te_3_ phase ($R3\bar{m}$, JCPDS No. 15‐0874). No noticeable appearance of Te phase was observed. The average thickness of the plate is determined as 10 nm by the atomic force microscopy analysis, with lateral size around 1–1.5 µm (Figure S2, Supporting Information). This highly anisotropic growth is derived from the inherent crystal structure of the material.^8^ The as‐fabricated Sb_2_Te_3_ nanoplates were cleaned by hydrazine hydrate and redispersed in a mixture of ethylene glycol (EG), glycerol, and ethanol with optimized ratio of 35:5:60 wt%. The remaining polyvinylpyrrolidone (PVP) surfactant helps prevent the nanoplates from aggregation, which can be removed in the following sintering process.

Figure 1c describes the schematic of 3D conformal aerosol jet printing process using solution‐processed nanostructures as building blocks, such as Sb_2_Te_3_ nanoplates. More printing details were provided in the Experimental Section and Table S1 (Supporting Information). Figure 1d gives the optical image of the synthesized ink (concentration ≈20 wt% of inorganic particles) with high stability that is printable for 3 months. Using the colloidal nanocrystal ink, TE films with virtually any patterns can be produced by the present method onto both flat and curved substrates. The film thickness can be adjusted by controlling the ink concentration, the mass flow rate, and number of printing passes. For example, a dense and continuous Sb_2_Te_3_ thin film (thickness around 1.5 µm) was printed on polyimide (Figure 1e), with competitive internal resistance while maintaining excellent flexibility. Figure 1f shows the corresponding SEM image after sintering, revealing well‐connected Sb_2_Te_3_ networks that facilitate efficient charge carrier transport across neighboring nanoplates. Films printed on various substrates were obtained (Figure 1g–i), including patterns with different designs, and a thin‐film printed on a cylindrical tube, demonstrating the promising ability of the present 3D conformal printing approach.

**Figure 2:**
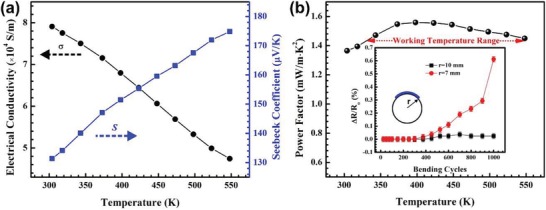
Temperature‐dependent in‐plane TE properties, including a) electrical conductivity and Seebeck coefficient, and b) power factor of flexible Sb_2_Te_3_ film printed on kapton after sintering at 400 °C. A high power factor over a wide temperature range (350–550 K) was presented. The inset in (b) shows the excellent flexibility of the film under repeated bending tests, with *R*_0_ the original resistance of the film and *r* the bending radius.

Thermal sintering plays an important role in removing the surfactant and consolidates the loose nanoparticle assembly into a densified continuous network. Here, a temperature of 400 °C was used in order to decompose and remove the remaining PVP surfactant. **Figure** **2**a shows the temperature‐dependent electrical conductivity σ and Seebeck coefficient *S* measured along the in‐plane direction of the sintered film. The film shows degenerate semiconducting behavior of decreasing σ with temperature, which is typical for Sb_2_Te_3_ nanostructures after sintering.^45^ The σ is lower than that of the single‐crystal bulk counterpart due to the electron scattering at the nanograin boundaries. Nevertheless, the room‐temperature σ reaches 7.8 × 10^4^ S m^−1^, which is among the highest reported values for printed TE films.^46^, ^47^ The carrier concentration (*p*) and mobility (*µ*) were determined by Hall measurement to be 1.01 × 10^20^ cm^−3^ and 48.5 cm^2^ V^−1^ s^−1^, respectively. The high carrier concentration is in agreement with the Te‐deficient defects as verified by energy‐dispersive X‐ray spectroscopy (EDS) analysis (Figure S3 and Table S2, Supporting Information), similar as previous study.^45^ The Seebeck coefficient *S* also shows degenerate semiconductor behavior, i.e., *S* increases with increasing temperature. The room‐temperature *S* of 130 µV K^−1^, which is similar to that of the other Sb_2_Te_3_‐based nanostructures,^31^ is over 30% higher than that of the single‐crystal bulk counterpart.^26^ The temperature‐dependent PF is shown in Figure 2b, demonstrating a competitive value around 1.37 mW m^−1^ K^−2^ at room temperature. It is noteworthy that the PF shows an average value of 1.5 mW m^−1^ K^−2^ over a wide temperature plateau (350–550 K), which is promising for harvesting waste heat which is abundant in the low‐to‐medium temperature range. The flexibility of the film was also studied using repeated bending testing under different curvatures. A slight increase of 0.6% of the electrical resistance was observed over 1000 bending cycles with curvature radius around 7 mm, demonstrating excellent flexibility of the printed film.

**Figure 3:**
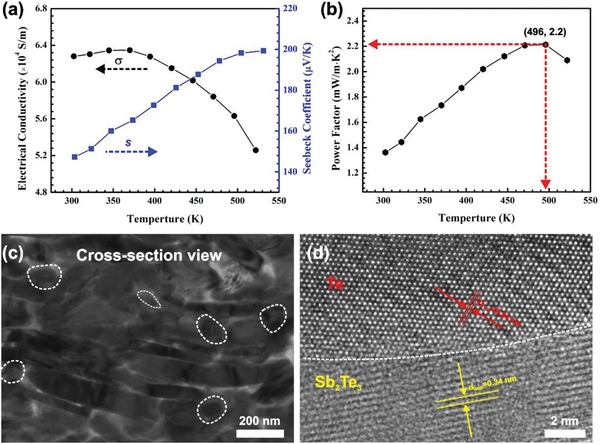
Temperature‐dependent in‐plane TE properties, including a) electrical conductivity and Seebeck coefficient, and b) power factor of the flexible Sb_2_Te_3_‐Te film printed on polyimide after sintering at 400 °C. c) TEM and d) HRTEM images of the interface between Te and Sb_2_Te_3_ from cross‐plane viewpoint of the printed film. The marked dashed circles imply the existence of additional Te nanorods, with local EDS and element line‐scan analysis given in the Supporting Information.

To further increase the TE performance, a nanocomposite of mixed 2D Sb_2_Te_3_ plates and 1D Te nanorods was investigated, with fabrication details given in the Experimental Section. When the atomic percent of Te precursor was over 60 at%, the crystalline Te was generated as an additional phase beyond Sb_2_Te_3_. XRD characterization of the nanocomposite was given in Figure S4 (Supporting Information). In this case, several Te peaks were observed beyond XRD pattern of Sb_2_Te_3_. About 8 wt% of excess tellurium was found in the form of tellurium nanorods based on EDS analysis and SEM characterization (Figure S3 and Table S2, Supporting Information). Related temperature‐dependent TE properties are presented in **Figure** **3**a,b. The σ of the Sb_2_Te_3_‐Te film decreases with the increasing temperature above 350 K, showing a metallic electron–phonon scattering dominated behavior similar to the above pure Sb_2_Te_3_ film. The nearly flat but slightly increasing σ with temperature below 350 K is likely due to the lower carrier concentration in the Sb_2_Te_3_‐Te composite compared to that in the reference Sb_2_Te_3_, so that scattering processes (e.g., boundary scattering associated with the numerous interfaces) other than electron–phonon scattering become important in this temperature range. The room‐temperature *S* shows a 13.1% increase from 130 to 147 µV K^−1^, and the room‐temperature PF maintains almost the same value as the pure Sb_2_Te_3_ film despite a small decrease of the σ with Te addition. The *S* of Sb_2_Te_3_‐Te nanocomposite film keeps increasing to about 200 µV K^−1^ at 525 K (Figure 3a), which is about 16.3% higher than that of the Sb_2_Te_3_ film (≈172 µV K^−1^). Meanwhile, the σ of the Sb_2_Te_3_‐Te film also surpasses that of the pure Sb_2_Te_3_ film when temperature exceeds 450 K. Comparison of the TE properties between Sb_2_Te_3_ and Sb_2_Te_3_‐Te films is given in Figure S5 (Supporting Information). As a result, the PF of the Sb_2_Te_3_‐Te film continues to increase monotonically and reaches a peak value of 2.2 mW m^−1^ K^−2^ at around 500 K, 46.7% higher than that of the Sb_2_Te_3_ film (Figure 3b). Comparison of TE performance of some representative p‐type flexible films around room temperature was listed in Table S4 (Supporting Information). Our composite‐based film can not only serve as power sources for flexible electronics around room temperature as the pure Sb_2_Te_3_ film, but also be used to recover waste heat in the low‐medium temperature range around 400–500 K.^44^

Transmission electron microscope (TEM) and high‐resolution TEM images of printed Sb_2_Te_3_‐Te film were given in Figure 3c,d. The Te precipitates are easily identified from the Sb_2_Te_3_ matrix from the lattice mismatch. As expected, the printed 1D/2D composite is composed of Sb_2_Te_3_ nanoplates and Te nanorods. The second phase Te appears as circular dots on the cross‐section of the focused ion beam (FIB) milled film. The local EDS analysis and element line‐scan (Table S3 and Figure S6, Supporting Information) confirmed the Te‐rich phase of >85 at% Te. Here, the larger nanorods are formed during the sintering process, similar as previous reports.^34^, ^48^ The room temperature carrier concentration and mobility of the Sb_2_Te_3_‐Te films were determined to be 4.95 × 10^19^ cm^−3^ and 79.3 cm^2^ V^−1^ s^−1^, respectively.

**Figure 4:**
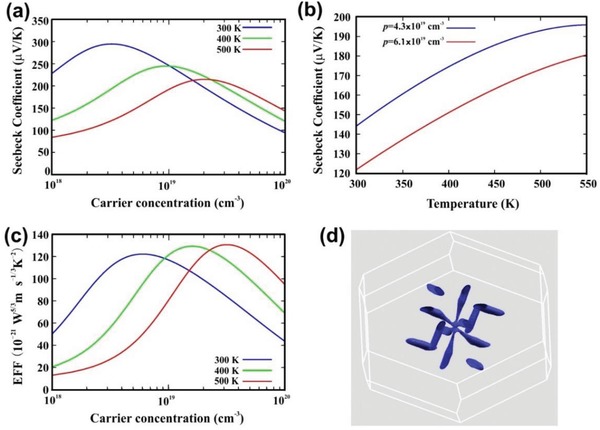
Transport properties from Boltzmann transport calculations and carrier pocket visualization for bulk Sb_2_Te_3_. a) Seebeck coefficient as a function of carrier concentration at 300, 400, and 500 K. b) Seebeck coefficient as a function of temperature for two fixed doping levels. c) Electronic fitness function (EFF). d) Carrier pocket visualization showing isosurfaces 0.1 eV below the valence band maximum. Here, the transport data are direction averages.

**Figure** **4**a shows the *S* obtained from Boltzmann transport calculations as a function of carrier concentration *p*. The *p* can be estimated by comparing experimental values with these curves. Figure 4b shows the calculated *S*(*T*) for two carrier concentrations, specifically 4.3 × 10^19^ cm^−3^ and 6.1 × 10^19^ cm^−3^. These values were chosen to be consistent with the ambient temperature experimental *S*. As can be seen, the curve for 4.3 × 10^19^ cm^−3^ is close to the experimental data of Figure 3a for Sb_2_Te_3_‐Te system, while the higher doping level curve compares well with the Sb_2_Te_3_ data in Figure 2a. Note that the *p* in these theoretical curves is the chemical carrier concentration, which is not the same as the Hall values due to the nonparabolic band structure. The similar temperature dependence of the calculated curves and the experimental data supports the inference that reduced doping level in the nanostructured Sb_2_Te_3_‐Te leads to enhancement in *S*. The calculated electronic fitness function (EFF), shown in Figure 4c, measures the decoupling of *S* and σ through band structure effects, and is closely related to the PF. Importantly, Sb_2_Te_3_ has a complex band structure, strongly affected by spin–orbit coupling, related to its topological insulator nature. This deviates from a single parabolic band model (Figure S7, Supporting Information). The EFF shows both elevated values and an increase as carrier concentration is reduced from typical values of the bulk. This leads to high values in the carrier concentration range where *S* is consistent with the experimental values for nanostructured Sb_2_Te_3_‐Te system.^49^ The EFF indicates that the nanostructured Sb_2_Te_3_‐Te is close to optimum doping at 500 K. It is also noteworthy that the calculated values of the EFF are quite high, reaching 1.3 × 10^−19^ W^5/3^m s^−1/3^ K^−2^ at 500 K. A value of 1.2 × 10^−19^ W^5/3^m s^−1/3^ K^−2^ is obtained at 300 K, albeit at lower carrier concentration. These values are comparable to some of the best TE materials.^49^ The high EFF is connected with the topological insulator behavior of Sb_2_Te_3_, which leads to a highly nonparabolic band structure. This can be seen in the carrier pocket visualization of Figure 4d. Here, there are multiple, highly anisotropic carrier pockets, favorable for superior TE performance, and very different from the single spherical section characteristic of the isotropic parabolic band model.

**Figure 5:**
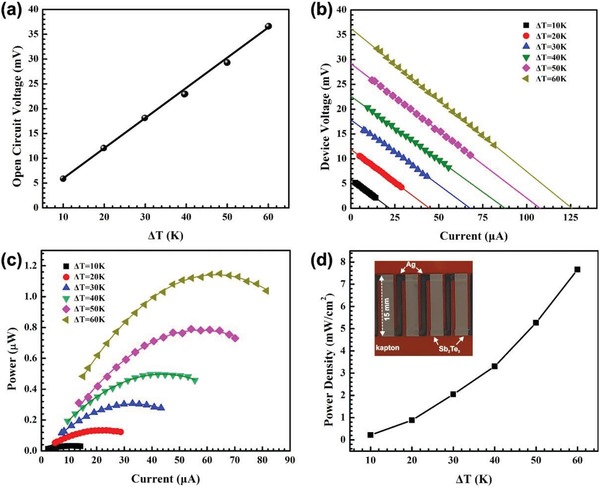
Performance of a flexible TE device fabricated by 3D conformal aerosol jet printing using optimized Sb_2_Te_3_‐Te and Ag as electrodes: a) experimental open circuit voltage (*V*_oc_) versus temperature differences (Δ*T*). b) device operating voltage versus current tested at various Δ*T*; c) power output versus electrical current at various Δ*T*; and d) experimental power density tested at various Δ*T* (Inset: photo of the printed device).

Finally, a flexible/wearable TE generator with aerosol jet printed Sb_2_Te_3_‐Te films and Ag electrodes was fabricated to demonstrate the printed *f*‐TEG for energy harvesting, as can be seen in **Figure** **5**. The output voltage and power show great promise for the development of small‐scale flexible/wearable TE generators, where the high PF of the printed TE films plays a significant role. Figure 5a shows that the measured device open circuit voltage (*V*_oc_) increases linearly with temperature difference (Δ*T*) by virtue of the Seebeck effect, achieving a maximum output voltage of 36.6 mV at Δ*T* of 60 K with only 4 TE elements. Figure 5b,c shows the device operating voltage and power output as a function of electrical current tested at different Δ*T*, respectively. A maximum power output of 1.15 µW was obtained with a Δ*T* of 60 K when the external load resistance matches the internal resistance of the device. The device power density increases with Δ*T* and reaches 7.65 mW cm^−2^ at Δ*T* of 60 K, as shown in Figure 5d. Here, the power density was evaluated based on the total cross‐sectional area of the four TE elements perpendicular to the heat flow direction. The high power density indicates that a small size of the printed *f*‐TEG is sufficient to power a range of typical internet of things and sensors.^50^

## 3 Conclusions

Creating functional materials with decent mechanical compliance while retaining competitive TE properties is a long‐standing challenge. In this paper, high‐performance and flexible thermoelectric films were produced by aerosol jet printing using solution‐processable nanostructures. The power factor of printed Sb_2_Te_3_ films reaches 1.37 mW m^−1^ K^−2^ at around 300 K, with a competitive average power factor lager than 1.5 mW m^−1^ K^−2^ from 350 to 500 K. In addition, a 1D/2D nanocomposite film printed using a mixture of 1D Te nanorods and 2D Sb_2_Te_3_ nanoplates shows an ultrahigh peak power factor of 2.2 mW m^−1^ K^−2^ at 500 K, which is especially attractive for waste heat recovery applications at medium‐temperature range. This is achieved by the control of carrier concentration near the optimum in the nanostructured composite film. A flexible thermoelectric generator was demonstrated, achieving a competitive device power density of 7.65 mW cm^−2^ with a temperature difference of 60 K. The aerosol jet printing technique not only provides enormous opportunities in scalable manufacturing of flexible TE devices for energy harvesting and cooling application, but also can be readily applied to explore other device architectures for broad applications beyond thermoelectrics.

## 4 Computational Section

### 4.1 Transport and Electronic Structure Calculations

Density functional calculations were performed for bulk Sb_2_Te_3_ with the Perdew–Burke–Ernzerhof Generalized Gradient Approximation.^51^ The calculations were done using the general potential linearized augmented planewave method as implemented in the WIEN2k code.^52^ Experimental lattice parameters were used, and the nonsymmetry constrained atomic positions were determined by total energy minimization. Transport calculations were done using the BoltzTraP code with the constant scattering time approximation.^53^ The electronic fitness function was then obtained from the transport data using the transM code (http://faculty.missouri.edu/singhdj/transm.shtml).

## 5 Experimental Section

*Nanoparticle Fabrication*: In a typical synthesis of pure Sb_2_Te_3_, 70 mL ethylene glycol (EG) solution containing mixed antimony trichloride (SbCl_3_, 6 mmol), tellurium dioxide (TeO_2_, 9 mmol), sodium hydroxide (NaOH, 1.5 g), and PVP (*M*_s_ ≈ 40 000 g mol^−1^, 0.8 g) were heated to 120 °C. 10 mL hydrazine hydrate (N_2_H_4_) was swiftly injected. The mixture was maintained at 130 °C for 30 min, then heated at 155 °C under reflux for another 10 h. The precipitates were collected by centrifugation at 5000 rpm, washed using ethanol for another three times. To fabricate the Te‐rich nanocomposite, a nominal 10 at% excess of TeO_2_ was added with the other conditions remain the same. In the case when Te precursor was over 60 at%, additional Te was generated as a second phase.

*Ink Preparation and Aerosol Jet Printing*: The as‐fabricated nanoplates were dispersed in solution to form a stable ink to allow aerosol jet printing (OPTOMEC AJP 300) using a pneumatic atomizer. Normal inkjet printing has a strict requirement of viscosity of 5–15 cp. Aerosol jet printing, on the other hand, can print ink with viscosity from 1 to 1000 cp with different nanostructures, providing a versatile approach with high spatial resolution. The spatial resolution of the aerosol jet printing can reach ≈20 µm in lateral dimension and several hundred nanometers in film thickness. In this work, the ink composition was optimized to achieve optimal film deposition and all‐printed TE devices. The solvent of Sb_2_Te_3_ ink was a mixture of EG, glycerol, and ethanol with optimized composition of EG: Glycerol: Ethanol 35:5:60 wt%. After that, Sb_2_Te_3_ particles with weight ratio of 20% were added, followed by a strong probe sonication (20 min) and bath sonication (30 min).

*Sintering of As‐Printed Film*: After cold press under 15 MPa for 15 min, thermal sintering was performed. The films were preheated at 110 °C for 1.5 h to remove remaining solvent under N_2_, and then sintered at 400 °C for 1 h with an increasing rate of 1.5 °C min^−1^, then decreased to 250 °C and hold for another 2 h. Finally, the samples were cooled down to room temperature.

*Characterizations*: The synthesized Sb_2_Te_3_ nanoplates were analyzed by XRD using Cu Kα radiation (λ = 1.5418 Å, Bruker D2 Phaser). TEM and High Resolution TEM techniques including the selected area electron diffraction images were performed using a JEM‐2100 electron microscope. TEM specimens were prepared using FIB milling process, a FEI Helios FIB‐SEM (FEI Company, Hillsboro, OR, USA) was used for serial sectioning and data collection. The morphology of all films (cross section and top‐view) was measured by the Scanning Electron Microscope Magelllan 400 (FEI Company), with working voltage 15 KV and working distance 4.5 mm.

*Thermoelectric Properties Measurement*: Seebeck coefficient was measured with a transient method wherein the temperature difference was increased from 1 to 5 K at ≈0.5 K min^−1^. Electrical conductivity was measured before Seebeck coefficient when temperatures were held at steady state with less than 0.05 K min^−1^ change in absolute temperature and in temperature difference. More details about the measurement setup and method were given in Figure S8 (Supporting Information).

## Conflict of Interest

The authors declare no conflict of interest.

## Supporting information

Supplementary
