# Supplementary material for: 3D Printing of Solution‐Processable 2D Nanoplates and 1D Nanorods for Flexible Thermoelectrics with Ultrahigh Power Factor at Low‐Medium Temperatures
Source: Adv Sci (Weinh). 2019 Oct 14;6(23):1901788. doi: 10.1002/advs.201901788 (PMC6891908; doi:10.1002/advs.201901788)
Supplement: Supplementary file 1 — Supplementary [file ADVS-6-1901788-s001.pdf]

Copyright WILEY-VCH Verlag GmbH & Co. KGaA, 69469 Weinheim, Germany, 2019.

ADVANCED SCIENCE
Open Access

Supporting Information

for *Adv. Sci.,* DOI: 10.1002/advs.201901788

3D Printing of Solution-Processable 2D Nanoplates and 1D Nanorods for Flexible Thermoelectrics with Ultrahigh Power Factor at Low-Medium Temperatures

*Chaochao Dun, Wenzheng Kuang, Nicholas Kempf, Mortaza Saeidi-Javash, David J. Singh, and Yanliang Zhang**

Supporting Information

3D Printing of Solution-Processable 2D Nanoplates and 1D Nanorods for Flexible Thermoelectrics with Ultrahigh Power Factor at Low-Medium Temperatures

*Chaochao Dun, Wenzheng Kuang, Nicholas Kempf, Mortaza Saeidi-Javash, David J. Singh, and Yanliang Zhang**

Dr. C. Dun, W. Kuang, N. Kempf, M. Saeidi-Javash, Prof. Y. Zhang

Deapartment of Aerospace and Mechanical Engineering, University of Notre Dame, Notre Dame, Indiana 46556, USA

E-mail: yzhang45@nd.edu

Prof. D. J. Singh

Department of Physics and Astronomy, University of Missouri, Columbia, Missouri 65211, USA

Results and Discussion

Table S1. Printing parameters of TE films using Sb_2Te_3 ink, or hybrid Sb_2Te_3-Te ink.

Materials	Parameters	Values
Sb_2Te_3 ink or Sb_2Te_3-Te ink	Nozzle diameter (μm)	150
	Line spacing (μm)	15
	PA atomizer flow (ccm)	550
	Exhaust flow (ccm)	500
	Sheath gas flow (ccm)	12
	Platen temperature (°C)	70
	Process speed (mm/s)	5

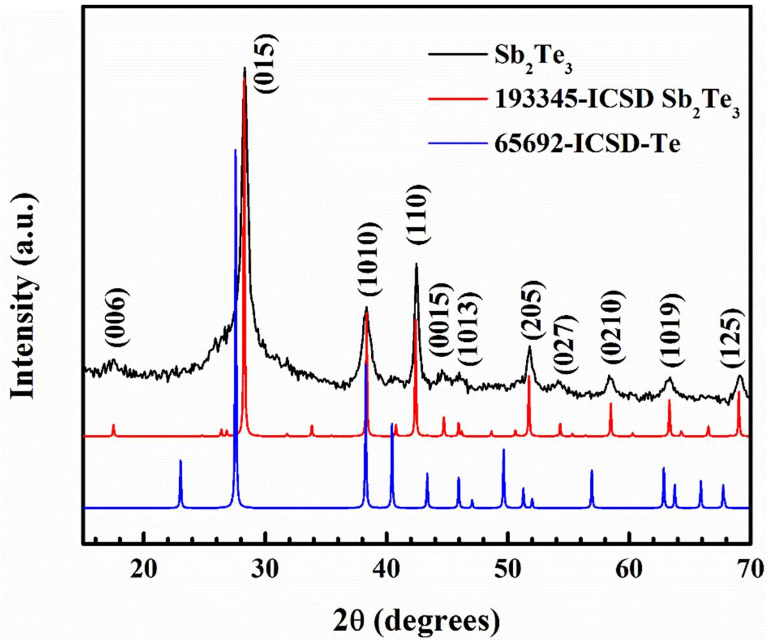

Figure S1. Powder X-ray diffraction (XRD) characterization of pure Sb_2Te_3 product without sintering. The standard XRD patterns for Sb_2Te_3 and Te were also given for comparison. The peaks corresponding perfectly to the rhombohedral Sb_2Te_3 phase as labelled by the Miller indexes. No noticeable appearance of Te phase was observed.

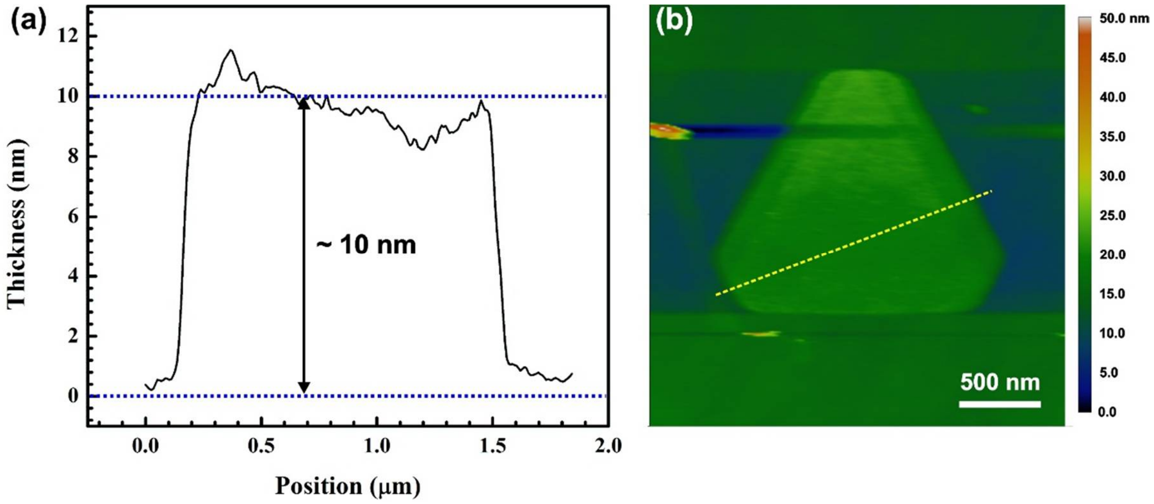

Figure S2. Atomic force microscopy (AFM) characterization of pure 2D Sb_2Te_3 plate. The average thickness of the plate is determined as 10 nm.

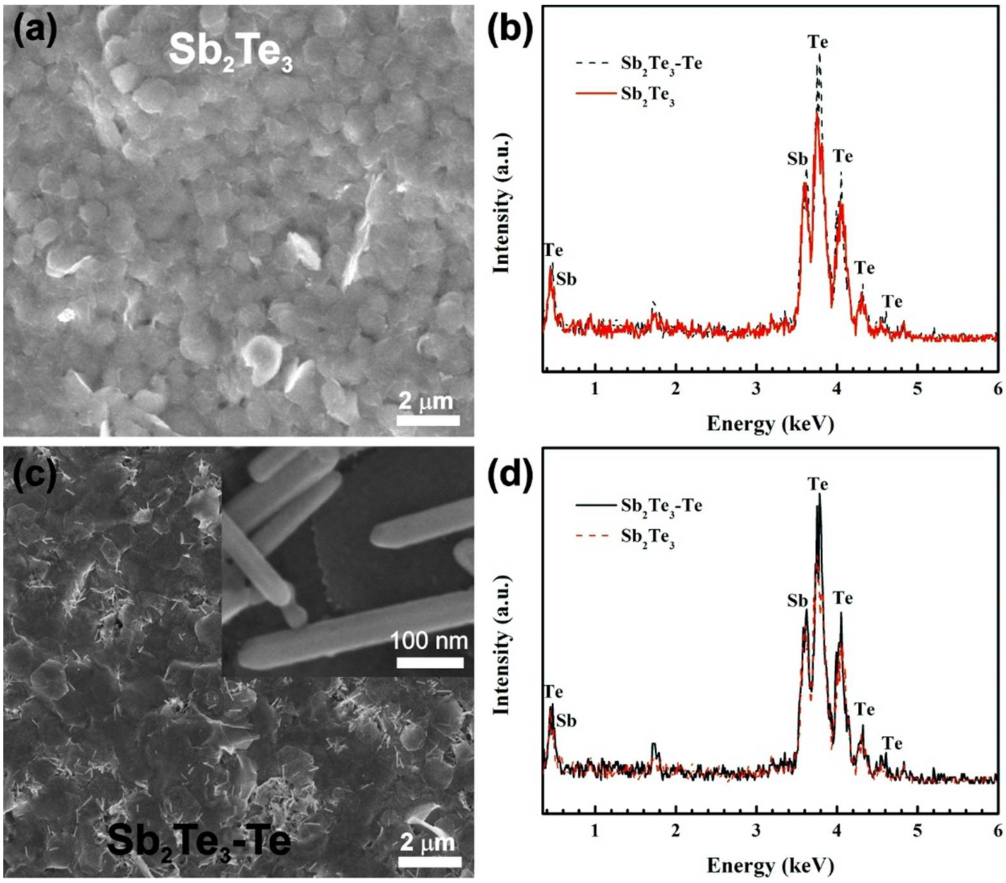

Figure S3. Energy-dispersive X-ray spectroscopy (EDS) characterization of pure Sb_2Te_3 and Sb_2Te_3-Te films. The corresponding element ratio was given in **Table S2**, which shows the Te-rich composite has about 8 wt% Te higher than the standard stoichiometric ratio of pure Sb_2Te_3.

Table S2. EDS analysis of pure Sb_2Te_3 and Sb_2Te_3-Te films after sintering.

Samples	Element	Weight %	Atomic %
**Sb_2Te_3- Te**	Sb	34.9	36.0
	Te	65.1	64.0
**Sb_2Te_3**	Sb	40.7	41.8
	Te	59.3	58.2

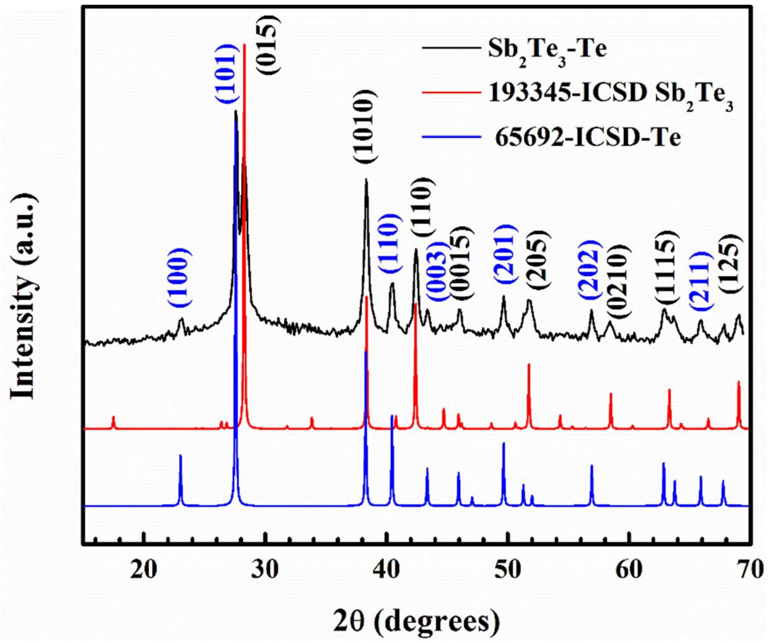

Figure S4. Powder X-ray diffraction (XRD) characterization of Sb_2Te_3-Te product without sintering, showing the appearance of trigonal Te phase. The standard XRD patterns for Sb_2Te_3 and Te were also given for comparison.

It is well known that the formation mechanism of nanostructure Sb_2Te_3 is,[1]

$$TeO_2+N_2H_4=Te+N_2\uparrow+2H_2O \quad (1)$$

$$2Te+N_2H_4+4OH^-=2Te^{2-}+N_2\uparrow+4H_2O \quad (2)$$

$$2Sb^{3+}+3Te^{2-}=Sb_2Te_3 \quad (3)$$

i.e. TeO_2 are firstly reduced to Te^{2-} ions with metal ions (Sb^{3+}) remain unchanged, after which Te^{2-} and Sb^{3+} react to generate the telluride product. It means 1D-Te nanostructures, which are intermediary product, act as template during the early stage of the fabrication. Sb_2Te_3 with 2D plates-like morphology will be developed if 1D rod-like Te template was destroyed in step (2) under alkaline condition at proper temperature (~155 °C); Otherwise, Te with 1D rods-like morphology will be preserved with excess Te precursor, which is consistent with previous report.[2,3] Therefore, by appropriately controlling the amount of reducing agent ($N_2H_4{\cdot}H_2O$) and the precursors TeO_2, pure 2D Sb_2Te_3 and Te-rich 1D/2D Sb_2Te_3-Te composites can be obtained.

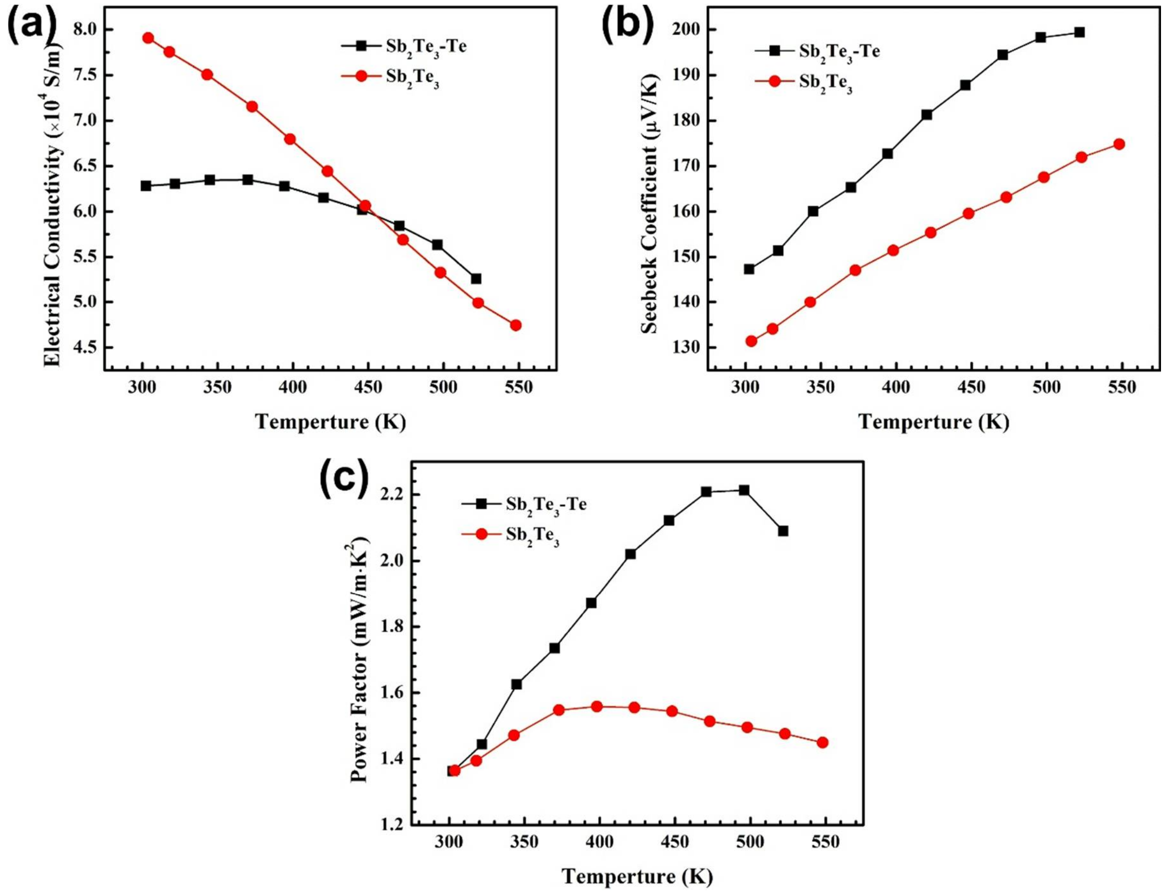

Figure S5. Comparison of TE performance between Sb_2Te_3 and 8 wt% Te-rich Sb_2Te_3-Te films, including (a) electrical conductivity, (b) Seebeck coefficient and (c) power factor.

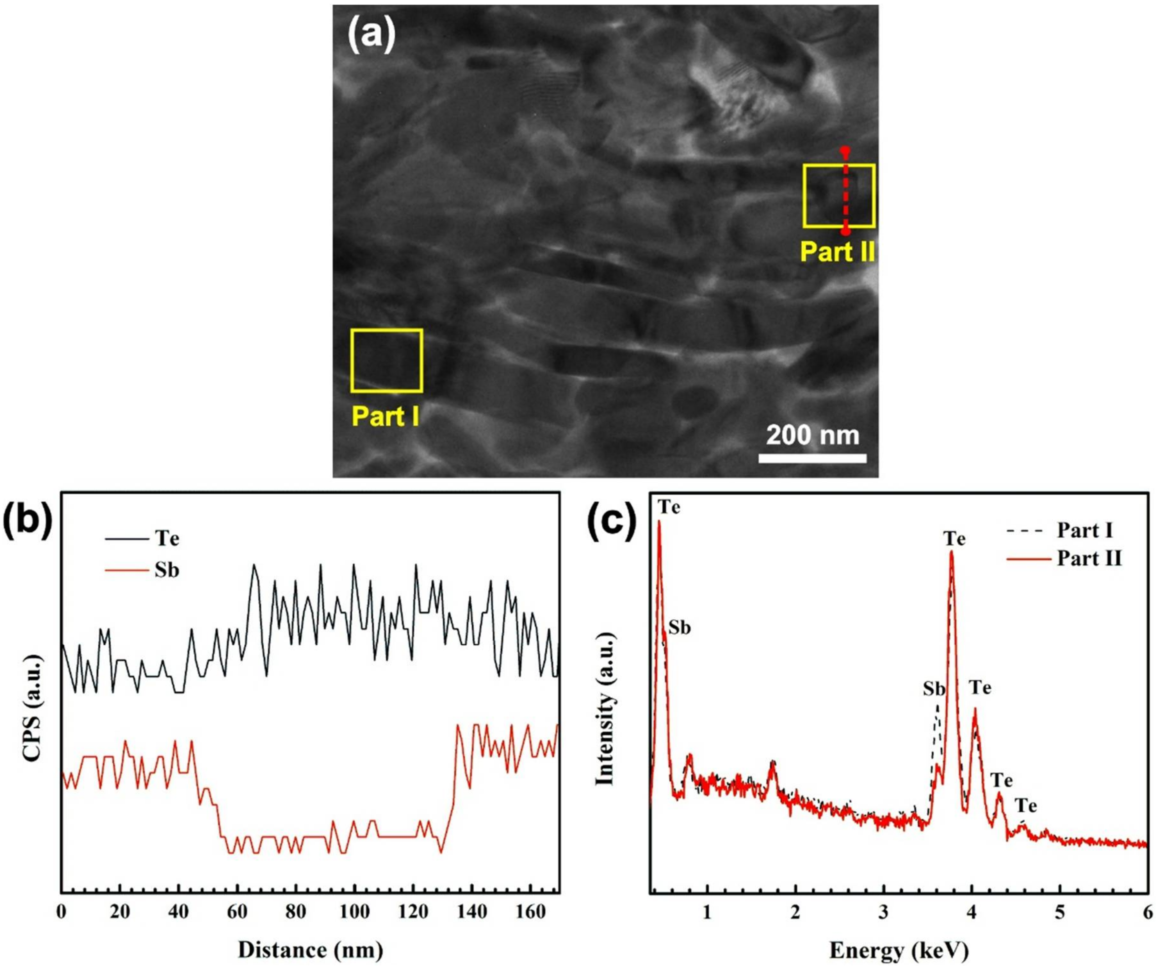

Figure S6. Local EDS Sb_2Te_3-Te films at different positions corresponding to **Figure 3(e)**. Related element ratio analysis was given in **Table S3**.

Table S3. Local EDS analysis of Sb_2Te_3-Te films after sintering.

Position	Element	Weight %	Atomic %
**I**	Sb	40.4	41.5
	Te	59.6	58.5
**II**	Sb	14.7	15.3
	Te	85.3	84.7

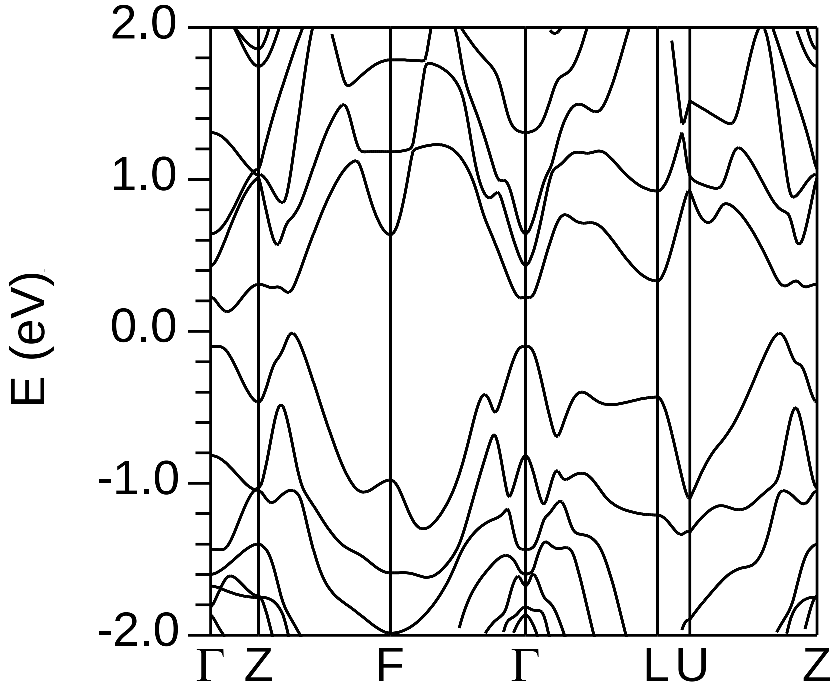

Figure S7. Band structure of Sb_2Te_3 showing a single parabolic band (SPB) model.

Table S4. Comparison of TE performances of *p*-type flexible films around room temperature.

**Materials**	σ (10^4 S m^{-1})	S (μV K^{-1})	$S^2\sigma$ (mW m^{-1} K^{-2})
PEDOT/Bi_2Te_3[4]	4.8	168	~1.35
Ag-Sb_2Te_3[5]	3.5	103	~0.37
$Bi_{0.5}Sb_{1.5}Te_3$[6]	0.2	278	~0.18
PANI/Te[7]	1.2	93	~0.10
CuI/PET[8]	1.3	172	~0.38
Doped CNT[9]	~17	~20	~0.7
$Ca_3Co_4O_9$[10]	~1.5	~125	~0.23
This work			
Sb_2Te_3-Te	6.3	147	~1.36

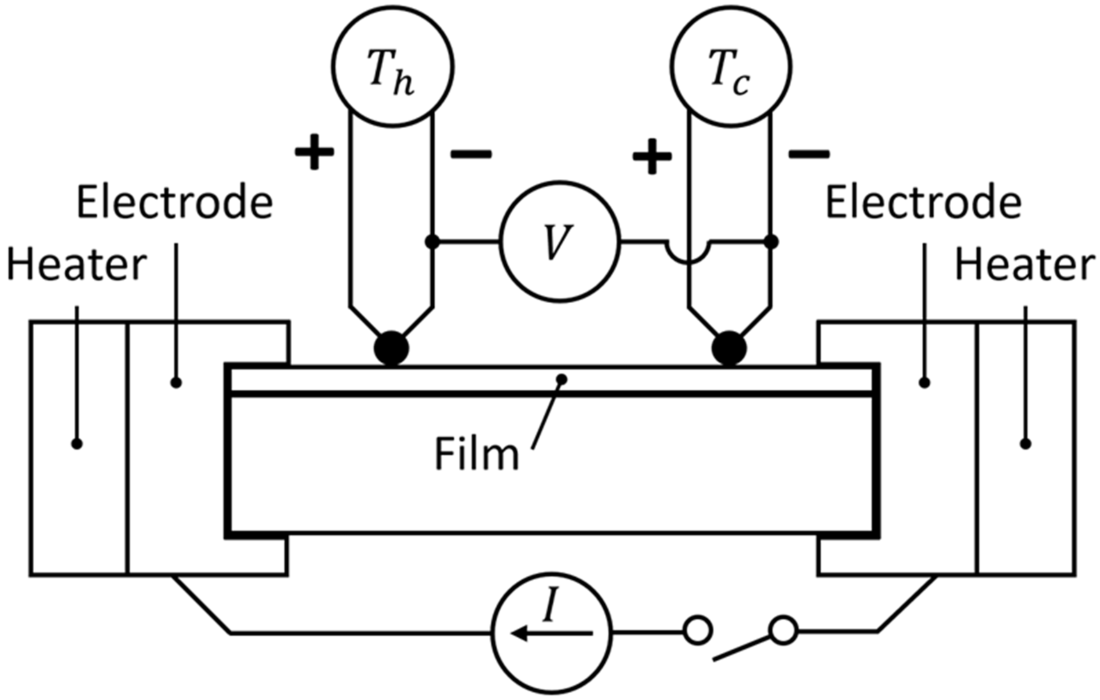

Figure S8. Experimental setup (cross-section view) for measuring thin film Seebeck coefficient and electrical conductivity.

Seebeck coefficient and electrical conductivity are measured at the same time and temperature using a home-built setup. The film is held to a dielectric support substrate with two electrodes through which current I is supplied for electrical conductivity measurement. Two thermocouples are brought into contact with the film and the measurement apparatus placed in a helium environment to improve thermal contact between the thermocouple tips and the film. On the other side of the electrodes are gradient heaters allowing precise control over the temperatures T_h and T_c and thus the temperature gradient $\Delta T = T_h - T_c$. Before measurement begins, the temperature gradient across the film is prescribed to be ΔT=1K. When all temperatures are at steady state (less than 0.05 K/min change in absolute temperature and in temperature gradient ΔT) the current switch is closed and current is sent through the sample. The voltage is measured across the negative leads of the thermocouples. This is done for five equally spaced currents. The sample resistance R is the slope of the best fit line through the V/I data and the conductivity is then calculated as $\sigma = L/RA$, where L is

the distance between the thermocouple tips and A is the cross-sectional area of the sample. Seebeck coefficient measurement begins by opening the current switch and slowly ramping the temperature gradient ΔT from 1 K to about 5 K while recording ΔT and voltage difference ΔV across the negative leads of the thermocouples with alternating measurements equally spaced in time. The negative of the slope of the best fit line through the ΔV/ΔT data gives the Seebeck coefficient relative to the material comprising the negative lead of the thermocouple. The absolute Seebeck coefficient of the sample is obtained by adding the absolute Seebeck coefficient of the negative thermocouple wire material to the measured sample relative Seebeck coefficient. The measurement error of the home-built set-up was less than 2% for the electrical conductivity and less than 3% for the Seebeck coefficient, respectively. The instrument was calibrated using a standard constantan sample of known properties.

Reference

[1] W. Shi, L. Zhou, S. Song, J. Yang, H. Zhang, *Adv. Mater.* **2008**, *20*, 1892.

[2] G.-H. Dong, Y.-J. Zhu, L.-D. Chen, *CrystEngComm* **2011**, *13*, 6811.

[3] Y. Liu, Y. Zhang, S. Ortega, Ibáñez， Maria, K. H. Lim, A. Grau-Carbonell, S. Martí-Sánchez, K. M. Ng, J. Arbiol, M. V. Kovalenko, D. Cadavid, A. Cabot, *Nano Lett.* **2018**, *18*, 2557.

[4] L. Wang, Z. Zhang, Y. Liu, B. Wang, S. Wang, *Nat. Commun.* **2018**, *9*, 3817.

[5] C. Dun, C. A. Hewitt, Q. Li, J. Xu, D. C. Schall, H. Lee, Q. Jiang, D. L. Carroll, *Adv. Mater.* **2017**, *29*, 1700070.

[6] D. Madan, Z. Wang, A. Chen, P. K. Wright, J. W. Evans, *ACS Appl. Mater. Interfaces* **2013**, *5*, 11872−11876.

[7] Y. Wang, S. M. Zhang, Y. Deng, *J. Mater. Chem. A Mater. energy Sustain.* **2016**, *4*, 3554.

[8] C. Yang, D. Souchay, M. Knei?, M. Bogner, H. M. Wei, M. Lorenz, O. Oeckler, G. Benstetter, Y. Q. Fu, M. Grundmann, *Nat. Commun.* **2017**, *8*, 16076.

[9] B. A. Macleod, N. J. Stanton, I. E. Gould, D. Wesenberg, R. Ihly, Z. R. Owczarczyk, K. E. Hurst, C. S. Fewox, C. N. Folmar, H. Hughes, B. L. Zink, L. Blackburn, A. J. Ferguson, *Energy Environ. Sci.* **2017**, *10*, 2168.

[10] B. Paul, E. M. Bjo, A. Kumar, J. Lu, P. Eklund, *ACS Appl. Energy Mater.* **2018**, *1*, 2261.
